# The Relationship Between Psychological Capital and Teacher Career Commitment in Ethnic Areas of China: The Mediating Effects of Gratitude and Career Well-Being

**DOI:** 10.3389/fpsyg.2021.818274

**Published:** 2022-01-27

**Authors:** Dong Hu, Tianmei Zhou, Kaiji Zhou, Fang Deng

**Affiliations:** ^1^School of Psychology, Sichuan Normal University, Chengdu, China; ^2^Sichuan Top IT Vocational Institute, Chengdu, China; ^3^School of Literature and Journalism, Leshan Normal University, Leshan, China

**Keywords:** ethnic regions, teachers in primary and secondary schools, psychological capital, gratitude, career well-being, career commitment

## Abstract

The purpose of this study is to investigate the characteristics, relationships and mechanisms underlying the psychological capital, career commitment, gratitude and career well-being of teachers in ethnic areas. In total, 573 primary school and secondary school teachers in Sichuan Province (including 402 teachers in ethnic regions and 171 teachers in non-ethnic areas) were investigated. Following questionnaires were used to investigate these questions: “Psychological Capital Questionnaire for Primary and Secondary School Teachers,” “Gratitude Questionnaire,” “Teacher Career Well-being Questionnaire” and “Career Commitment Questionnaire for Primary and Secondary School Teachers.” The results show that the psychological capital of teachers in ethnic areas is higher than that of teachers in non-ethnic areas. Teachers in ethnic areas have lower levels of career well-being and lower levels of gratitude than teachers in non-ethnic areas. There was no significant difference in career commitment between teachers in ethnic areas and teachers in non-ethnic areas. There were significant positive correlations among psychological capital, gratitude, career well-being and career commitment. Psychological capital can predict career commitment significantly and positively. The mediating effect of career well-being between teachers’ psychological capital and career commitment was significant in both ethnic areas and non-ethnic areas. The chain of mediating effects between gratitude and career well-being was significant in non-ethnic areas. In conclusion, psychological capital can predict teacher career commitment effectively, and the prediction mechanism in ethnic areas is different from that in non-ethnic areas.

## Introduction

In recent years, as a result of the development of education in ethnic areas^1^ of China, the scale of schooling and quality of education in ethnic areas have significantly improved, which is a fact that has also necessitated higher requirements for the teaching force in ethnic areas. However, due to the limitations of living conditions, working conditions and economic treatment in ethnic areas, some teachers exhibit negative work attitudes, a sense of powerlessness with regard to their work and a lower sense of career well-being (Liu, 2017; Yang and You, 2017; Ma, 2018; Shao et al., 2018); this situation is likely to have a negative impact on the career commitment of those teachers.

Teacher career commitment refers to teachers’ level of reluctance toward career change due to their career identification, emotional attachment, career commitment, and internalization of social norms (Meyer et al., 1993; Wang et al., 2015). Teachers’ career commitment not only affects teachers’ career and It also affects teachers’ job performance and job satisfaction, which in turn affects the stability of the teaching force (Dang et al., 2020). Teacher career commitment is important for teachers’ career development and is directly related to teachers’ work engagement and teaching quality. Therefore, it is particularly important to understand the status of teachers’ career commitment in ethnic areas and to identify individual resources that can promote career commitment.

As an important psychological resource influencing employees’ organizational behavior, psychological capital may be an important factor in promoting career commitment among teachers in ethnic areas. Psychological capital is a higher-order concept based on positive psychological elements such as self-efficacy, optimism, hope, and resilience and it is a “state-like” psychological quality that reflects individual motivation (Luthans et al., 2006). Psychological Capital is concerned with bringing improvement within an individual both personally and professionally (Tang, 2020), psychological capital, as an important positive psychological resource, could positively affect emotional labor and vocational well-being (Zhao and You, 2021). The internal motivation of individuals can be established by examining their perception of their own abilities, as suggested by Bandura and Locke (2003). According to psychological capital theory, psychological capital can facilitate the integration of various internal, psychological resources but also other resources, such as physical resources, intellectual resources and interpersonal resources (Fredrickson and Branigan, 2005). This integration, in turn, facilitates the enhancement of internal motivational processes mentioned above, thus effectively increasing practitioners’ level of career commitment. It has been shown that psychological capital is significantly and positively related to employees’ career commitment (Singhal and Rastogi, 2018; Ye et al., 2019). However, few existing studies have explored this issue in relation to teacher groups in ethnic areas of China. The relationship between psychological capital and career commitment among teachers in ethnic areas has yet to be verified. Therefore, this study examined the relationship between psychological capital and career commitment among teachers in ethnic areas and proposed Hypothesis 1 as follows: the psychological capital of teachers in ethnic areas can significantly and positively predict their career commitment.

In addition to examining the predictive role that the psychological capital of teachers in ethnic areas plays with respect to their career commitment, this study also sought to examine the intrinsic mechanisms of action operating in this relationship. First, psychological capital may be able to act on career commitment through career well-being. According to the job-demand model and psychological capital theory, psychological capital may complement the depletion of intrinsic resources caused by job demands (Demerouti et al., 2001; Luthans et al., 2007) and help employees cope with difficulties and challenges more effectively in organizational situations and acquire more positive career attitudes, thereby enhancing their career well-being. It has been shown that psychological capital is significantly and positively related to teachers’ subjective well-being (Avey et al., 2010), job satisfaction (Bandura and Locke, 2003), and career happiness (Xu et al., 2020). Teachers’ career well-being reflects the ongoing positive experiences that teachers undergo when their needs are fulfilled through educational activities. According to the expanded construct theory of positive emotions (Fredrickson, 2001), as an enduring positive experience, teachers’ career well-being has not only a transient expanded function but also a long-term constructed function. In other words, career well-being provides enduring physical, cognitive, and social resources, thus contributing to enhanced career identity, increased career engagement, and improved performance. Because career identity is crucial to career commitment, career well-being can enhance teachers’ career commitment through career identity. It has been shown that teachers’ subjective well-being (Luo et al., 2006), life satisfaction (Kang, 2007), and career well-being (Wang, 2013) can significantly and positively predict career commitment. Therefore, this study proposed Hypothesis 2 as follows: teachers’ psychological capital in ethnic areas can indirectly act on career commitment through the mediating variable of career well-being.

Second, psychological capital may also act on teachers’ career commitment through gratitude. Gratitude is the psychological tendency of individuals to understand or respond with grateful cognitions, emotions, and behaviors to positive experiences or personal outcomes resulting from favors or help provided by others or by objects (McCullough et al., 2002). This tendency may be expressed in the context of the teaching profession or as practitioners’ gratitude for colleagues, leaders, organizations, and the profession as a whole. Thus, teachers with high gratitude tendencies may be more likely to develop gratitude toward school leaders and educational authorities, a development which enhances teachers’ identification with the profession and raises their sense of responsibility and obligation to the profession (Zhou et al., 2013), which in turn increases their level of career commitment. At the same time, psychological capital is likely to promote teachers’ gratitude tendencies; as mentioned above, psychological capital theory suggests that psychological capital is a dynamic psychological quality with constructive functions that can facilitate employees’ integration of various intrinsic psychological resources, and teachers’ gratitude tendencies, as an important psychological resource for their interpersonal interactions, are likely to be influenced by teachers’ psychological capital. It has been shown that there is a significant positive relationship between psychological capital and gratitude (Han et al., 2012; Zhang et al., 2013). Therefore, this study proposed Hypothesis 3 as follows: the psychological capital of teachers in ethnic areas can indirectly act on career commitment through the mediating variable of gratitude.

In addition, teachers’ gratitude tendency may also affect their career well-being. Although few studies have directly examined the relationship between employees’ gratitude and career well-being, there is still much indirect evidence for such a claim. It has been shown (Luo and Zhou, 2015; Liu et al., 2017) that gratitude has a significant positive correlation with subjective well-being. This result has been verified for different populations (Kang, 2007), e.g., the gratitude tendency of older adults is positively related to their subjective well-being and the gratitude tendency of secondary school students can significantly predict their subjective well-being. There are also meta-analyses showing that grateful individuals have stronger subjective well-being (Ding and Zhao, 2018). Therefore, teachers’ gratitude in ethnic areas may also positively predict their career well-being. As mentioned above, teachers’ psychological capital in ethnic areas may positively predict gratitude, which in turn may positively predict career well-being, from which it can be inferred that psychological capital in ethnic areas may enhance teachers’ career well-being through gratitude, which in turn may increase their level of career commitment. Therefore, this study proposed Hypothesis 4 as follows: the serial mediating effect of gratitude and career well-being on teachers’ psychological capital and career commitment in ethnic areas is significant.

**FIGURE 1:**
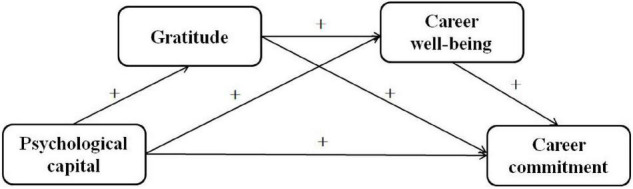
Schematic diagram of sequence mediation model.

In summary, this study examined the direct effect of the psychological capital of teachers in ethnic areas on their career commitment but also the mediating mechanisms of gratitude and career well-being (e.g., Figure 1). In addition, to examine whether the relationship between the psychological capital and career commitment of teachers in ethnic areas and the mechanisms of action underlying that relationship are unique, this study also selected a set of teachers from non-ethnic areas as a control sample.

## Materials and Methods

### Participants

The survey was conducted on primary and secondary school teachers in Sichuan Province, China. A total of 600 questionnaires were distributed, and 573 valid questionnaires were collected, including 402 teachers in the ethnic areas of Aba and Liangshan and 171 teachers in the non-ethnic areas of Dazhou and Neijiang; 300 male teachers and 273 female teachers; 404 Han teachers, 14 Tibetan teachers, 138 Yi teachers, 15 teachers from other ethnic groups, and 2 teachers whose ethnicity was unrecorded; 285 teachers with a bachelor’s degree or above (including 5 teachers with a graduate degree and 280 teachers with a bachelor’s degree), 288 teachers with a college degree or below, and 3 teachers whose educational status was unrecorded; and 486 teachers in the fields of language, mathematics and foreign affairs, 486 teachers in the fields of political science, history and geography, and 3 teachers whose area of teaching was unrecorded. Questionnaires were returned by 285 teachers (5 teachers with a graduate degree and 280 teachers with a bachelor’s degree), 288 teachers with a college degree or less, and 3 teachers whose educational data was unrecorded; 486 teachers in the fields of language, mathematics and foreign languages, 49 teachers in the fields of science, history, geography and chemistry, 30 teachers in the fields of music, sports and aesthetic education, and 8 teachers whose area of teaching was unrecorded; and 402 primary school teachers, 168 secondary school teachers (152 middle school teachers and 16 high school teachers), and 3 teachers whose level of teaching was unrecorded.

### Questionnaires

#### The Psychological Capital Questionnaire for Primary and Secondary School Teachers

This study used the Psychological Capital Questionnaire for Primary and Secondary School Teachers developed by Zhang (2010). The questionnaire contained 19 items, which were organized along 4 dimensions: resilience (6 items, e.g., “I will solve the difficulties I encounter in teaching no matter what”), self-confidence (4 items, e.g., “I believe I am capable of completing the teaching tasks assigned by the school”), optimism (5 items, e.g., “In my work, I always think in a positive direction concerning uncertain results”), and hope (4 items, e.g., “Currently, I am achieving the goal of being an excellent teacher”). The questionnaire was scored on a 7-point scale ranging from 1 = “strongly disagree” to 7 = “strongly agree,” with higher scores indicating higher levels of psychological capital. The validated factor analysis showed that *x*2/df = 4.32, GFI = 0.91, AGFI = 0.86, CFI = 0.88, NFI = 0.85, IFI = 0.88, and RMSEA = 0.08. The α coefficient of this questionnaire in the present study was 0.76, and the α coefficient of each dimension ranged from 0.60 to 0.62. This fact indicates that the questionnaire has acceptable letter validity in this study.

#### Career Commitment Questionnaire for Primary and Secondary School Teachers

This study used the Career Commitment Questionnaire for Primary and Secondary School Teachers developed by Liu (2005). The questionnaire contained 25 items, which are organized along 4 dimensions, namely, ideal value commitment (7 items, e.g., “My students’ success is my ideal”), obligation commitment (7 items, e.g., “I think teachers should not change jobs frequently”), opportunity cost (6 items, e.g., “If I were to choose again, I would not choose teaching as a career,” which was reverse scored), and commitment to realistic values (5 items, e.g., “Being a teacher will improve my ability to communicate and coordinate with others”). The questionnaire was scored on a 5-point scale ranging from 1 = “totally disagree” to 5 = “totally agree,” with higher scores indicating higher levels of career commitment. The validation factor analysis supported the structure of the questionnaire with *x*2/df = 2.44, GFI = 0.92, CFI = 0.91, NNFI = 0.90, and RMSEA = 0.05. The α coefficient of the questionnaire in this study was 0.85, and the α coefficients of the dimensions ranged from 0.65 to 0.76. This fact indicates that the questionnaire has good reliability and validity.

#### Teacher Career Well-Being Questionnaire

This study used the Teacher Career Well-being Questionnaire developed by Xu (2013). It contained 32 items, which were organized along 5 dimensions, namely, need for satisfaction (11 items, e.g., “My work is recognized and respected by society”), career identity (7 items, e.g., “I love teaching very much”), achievement (5 items, e.g., “I am very satisfied with the progress my students have made”), sense of value (4 items, e.g., “I can realize my own value in life by teaching”), and friendly experience (5 items, e.g., “My family is very supportive of my work”). The questionnaire was scored on a 5-point scale ranging from 1 = “not at all” to 5 = “completely,” with higher scores indicating higher levels of career well-being. The validation factor analysis supported the structure of the questionnaire with *x*2/df = 2.91, GFI = 0.73, CFI = 0.96, NFI = 0.95, NNFI = 0.96, and RMSEA = 0.09. The α coefficient of the questionnaire in this study was 0.94, and the α coefficients of the dimensions ranged from 0.71–0.92, indicating the high reliability of the questionnaire in this study.

#### Gratitude Questionnaire

This study used the Gratitude Questionnaire (GQ-6) developed by McCullough et al. (2002), and revised in 2011 by Wei et al. (2011) mainly to measure the tendency of individuals to identify feelings of gratitude. The questionnaire consists of 6 items, which were scored on a 6-point scale ranging from 1 = “strongly disagree” to 6 = “strongly agree,” with higher scores indicating greater gratitude identification. The questionnaire has demonstrated good reliability and validity in previous studies. In this study, the factor loadings of questions 3 and 6 of the questionnaire were too low, and the subjects did not understand the items correctly, so questions 3 and 6 of the original scale were deleted, and the final questionnaire entered the follow-up analysis with 4 items. In this study, the α coefficient of this questionnaire was 0.80.

### Data Collection Procedures and Processing

The questionnaire was administered to the subject group in the classroom of a teacher training class, and the time required was approximately 25 min. The main test was administered by a trained psychology graduate student, and before the questionnaire was administered, the main test subject read the instructions and the principles of anonymity and confidentiality for the test to the subjects. Microsoft Excel 2007, IBM SPSS 25.0, IBM AMOS 21.0, and Mplus 7.4 were used for statistical processing of the data.

### Control of Common Method Bias

Since this study used a questionnaire method to collect data and all data were obtained from subjects’ self-reports, common method bias may exist in the measurements. Common method bias is often controlled in two ways: procedural control and statistical control (Podsakoff et al., 2003). The procedural controls in this study were as follows: (1) appropriate variation of different questionnaire guidelines, scoring, and reverse scoring of one-third of questionnaire items; (2) use of anonymity and confidentiality for the questionnaire to ensure truthful responses; and (3) use of a uniform group administration questionnaire at the training course level. The statistical control was tested using Harman’s one-way test, and the results showed that a total of 15 factors in this study had an eigenroot value greater than one, and the variance explained by the first factor was 28.80%, which was less than the critical value of 40%, thus indicating that there were no serious problems resulting from common method bias in this study.

## Results

### Basic Information on the Psychological Capital, Career Well-Being, Gratitude and Career Commitment of Teachers in Ethnic Areas

Descriptive statistics were conducted for all teachers’ scores on psychological capital, gratitude, career well-being, and career commitment measures, and differences in each of the main variables were examined for demographic variables such as ethnicity, gender, and education. The results are presented in Tables 1–3. In addition, because there were not enough minority teachers from non-ethnic areas in this survey (*n* = 2), the differences in the main variables among teachers of different ethnicities were not compared in the analysis of non-ethnic areas.

**TABLE 1:** Differences in the psychological capital, career well-being, gratitude and career commitment of teachers in ethnic and non-ethnic areas.

Variables	Ethnic area			Non-ethnic areas			*t*	*P*	Cohen’s *d*
	*M*	*SD*	*n*	*M*	*SD*	*n*			
Psychological capital	4.12	0.62	402	4.02	0.55	171	1.85	0.065	0.17
Career well-being	3.83	0.61	402	4.01	0.58	171	–3.45	<0.001	–0.3
Gratitude	5.05	0.83	402	5.26	0.85	171	–2.81	<0.01	–0.26
Career commitment	3.66	0.56	402	3.63	0.4	171	0.74	0.459	0.06

*M, mean value; SD, standard deviation; n, sample size; t, T-value; P, P-value.*

**TABLE 2:** Basic information on the psychological capital, career well-being, gratitude and career commitment of teachers in ethnic areas.

Variables	Category	*N*	Psychological capital		Gratitude		Career commitment		Career well-being	
			*M*	*SD*	*M*	*SD*	*M*	*SD*	*M*	*SD*
Gender	Male	214	4.1	0.59	5.04	0.88	3.76	0.61	3.57	0.56
	Female	185	4.19	0.64	5.06	0.76	3.9	0.59	3.76	0.55
*t*			−2.12*		−0.81		−2.14*		−3.42*	
Academic qualifications	Bachelor’s degree or above	154	4.18	0.62	4.84	0.73	3.88	0.6	3.64	0.58
	Junior college or above	245	4.08	0.61	4.75	0.77	3.9	0.58	3.67	0.55
*t*			1.48		1.18		−0.45		−0.54	
School	Primary school	332	4.12	0.61	4.75	0.76	3.94	0.56	3.7	0.55
	Secondary school:	68	4.10	0.66	4.9	0.73	3.67	0.65	3.47	0.58
*t*			0.25		−1.45		3.39**		3.12**	
Subjects	Language, mathematics and foreign language	359	4.14	0.63	5.06	0.81	4.35	0.74	3.68	0.56
	Other subjects	24	4.01	0.52	5.04	0.87	4.41	0.42	3.37	0.6
	Music, sports, and art	11	3.96	0.45	5.06	0.71	4.32	0.44	3.48	0.42
*F*			0.84		0.01		4.63*		4.07*	

M, mean value; SD, standard deviation; n, sample size; t, T-value. *p < 0.05 and **p < 0.01.

**TABLE 3:** Basic information on the psychological capital, career well-being, gratitude and career commitment of teachers in non-ethnic areas.

Variables	Category	*N*	Psychological capital		Gratitude		Career commitment		Career well-being	
			*M*	*SD*	*M*	*SD*	*M*	*SD*	*M*	*SD*
Gender	Male	88	4.07	0.53	5.14	1	3.88	0.56	3.64	0.44
	Female	83	3.98	0.57	5.38	0.67	4.14	0.57	3.62	0.37
*t*			1.17		–0.81		–3.06*		0.31	
Academic qualifications	Bachelor’s degree or above	128	4	0.59	4.35	0.69	4.09	0.58	3.62	0.42
	Junior college or above	43	4.08	0.4	4.39	0.6	4.11	0.49	3.65	0.35
*t*			–0.76		–0.41		–0.28		–0.35	
School	Primary school	70	3.99	0.61	4.3	0.6	4.11	0.5	3.59	0.37
	Secondary school:	100	4.05	0.51	4.38	0.7	4.09	0.59	3.66	0.43
*t*			–0.74		–0.72		0.3		–1.07	
Subjects	Language, mathematics and foreign language	127	4.1	0.57	5.2	0.9	3.84	0.62	3.63	0.43
	Other subjects	25	3.92	0.28	5.44	0.64	3.43	0.72	3.61	0.31
	Music, sports, and art	19	3.64	0.51	5.42	0.72	3.9	0.27	3.63	0.29
*F*			6.83**		1.19		1.35		0.02	

M, mean value; SD, standard deviation; n, sample size; t, T-value. *p < 0.05 and **p < 0.01.

As seen from Table 1, the psychological capital of teachers in ethnic areas was slightly higher than that of teachers in non-ethnic areas, and the test results reached borderline significance (*p* = 0.065). The gratitude and career well-being of teachers in non-ethnic areas were significantly higher than those of teachers in ethnic areas (*p* < 0.01). Table 2 shows the results of teachers from ethnic areas for the main variables of this study, from which it can be seen that the psychological capital (*t* = −2.12, *p* < 0.05, Cohen’s *d* = −0.21), career well-being (*t* = −2.14, *p* < 0.05, Cohen’s *d* = −0.23), and career commitment (*t* = −3.42, *p* < 0.01, Cohen’s *d* = −0.34) scores of male teachers from ethnic areas were significantly lower than those of female teachers; the career well-being of primary school teachers in ethnic areas was significantly higher than that of secondary school teachers (*t* = −3.39, *p* < 0.001, Cohen’s *d* = −0.47); teachers who taught different subjects in ethnic areas had significant variations in career well-being (*F*_2,391_ = 4.49, *p* < 0.05, $\eta_{\text{p}}^{2}$ = 0.02) and career commitment (*F*_2,391_ = 4.08, *p* < 0.05, $\eta_{\text{p}}^{2}$ = 0.02) differed significantly, with teachers of political science, history, geography, science and chemistry having significantly higher career well-being than teachers of language arts, mathematics and physical education and aesthetics (*p* < 0.05) and teachers of language arts and mathematics having higher career commitment than teachers of political science, history, geography, science and chemistry (*p* < 0.01). Table 3 reflects the situation of teachers in non-ethnic areas. From Table 3, it can be seen that female teachers in non-ethnic areas had significantly higher career well-being than male teachers (*t* = −2.12, *p* < 0.01, Cohen’s *d* = −0.46). Furthermore, there were significant differences in psychological capital among different teaching subjects in non-ethnic areas (*F*_2,168_ = 6.83, *p* < 0.01, $\eta_{\text{p}}^{2}$ = 0.08), i.e., teachers of phonics, physical education, and aesthetics were significantly lower than teachers of language arts and mathematics (*p* < 0.01).

### Correlation Analysis of Psychological Capital, Gratitude, Career Well-Being and Career Commitment

Pearson product-difference correlation analysis was conducted on psychological capital, career well-being, gratitude and career commitment, and the results are shown in Table 4. From Table 4, it is clear that there are significant correlations among psychological capital, career well-being, gratitude and career commitment in both ethnic and non-ethnic areas. The results of the correlation analysis provide a feasible basis for the next step of investigating mediating effects.

**TABLE 4:** Correlation analysis.

Variables	1	2	3	4
(1) Psychological capital	1	0.30*	0.25**	0.21**
(2) Career well-being	0.46***	1	0.23**	0.59***
(3) Gratitude	0.35***	0.31***	1	0.36**
(4) Career commitment	0.56***	0.72***	0.26***	1

Below the diagonal line are teachers from ethnic areas (N = 402), and above the diagonal line are teachers from non-ethnic areas (N = 171). *p < 0.05; **p < 0.01; and ***p < 0.001.

### Structural Equation Model Analysis

#### Project Packaging

This study adopts the single-factor method as part of the balance method for packaging the gratitude questionnaire (Bian et al., 2007) to avoid the inflated measurement error of latent variables that degrade model fit since the gratitude questionnaire is unidimensional and the questions are highly homogeneous. The gratitude questionnaire was packaged into two-question combinations, and the question with the highest factor loadings was put into the package as the anchor item, followed by inclusion of the questions with the next highest loadings in reverse order to the direction of the balance, and the score of each question combination after packaging was the mean value of the questions in the package. They are not packaged here, and the mean value of each of their dimensions is taken as the observation of the variables since the psychological capital questionnaire and career commitment questionnaire include four dimensions and the career well-being questionnaire includes five dimensions.

#### Mediating Effects of Gratitude and Career Well-Being on Psychological Capital and Career Commitment

Because the dependent variables differed significantly in terms of teacher gender, school level, and teaching subject, these variables needed to be controlled for in the structural equation model. Gender (based on male), school level (based on primary school), and teaching subject (based on language, mathematics, and foreign language) were transformed into dummy variables with codings 0 and 1.

**FIGURE 2:**
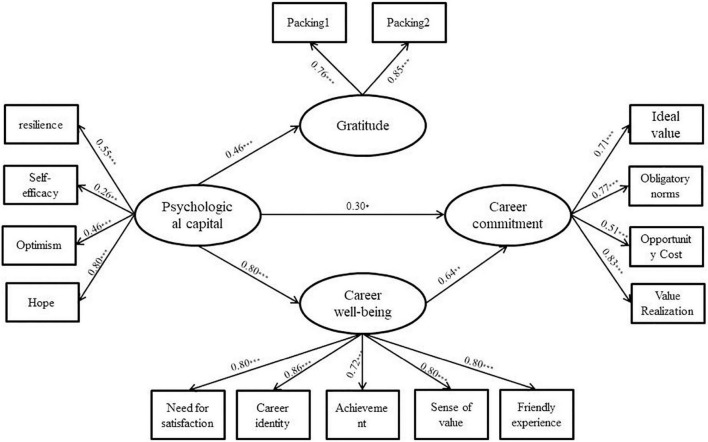
Structural equation test chart of the teacher mediation model in ethnic areas (modified model below). The model controls for gender, ethnicity, school level, and teaching subject, and the corresponding paths are not presented for the sake of the model’s simplicity and elegance. *p < 0.05; **p < 0.01; and ***p < 0.001.

**FIGURE 3:**
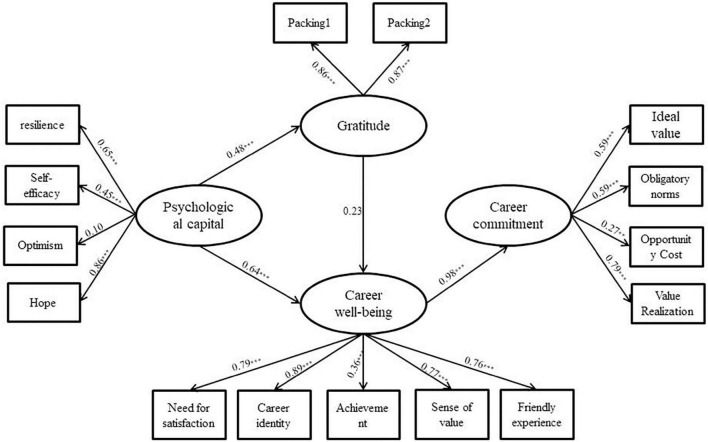
Structural equation test of the teacher mediation model in non-ethnic areas. *p < 0.05; **p < 0.01; and ***p < 0.001.

The regression coefficients of career commitment to gratitude (β = 0.04, *p* > 0.05, 95% CI = [−0.19, 0.16]) and career well-being to gratitude (β = 0.06, *p* > 0.05, 95% CI = [−0.05, 0.18]) for teachers in ethnic areas were too small and were insignificant; the regression coefficients of career commitment to psychological capital (β = 0.04, *p* > 0.05, 95% CI = [−0.23, 0.21]) and gratitude (β = 0.03, *p* > 0.05, 95% CI = [−0.16, 0.12]) had small regression coefficients with p values greater than 0.05 and 95% CI including 0, all of which were non-significant paths. Therefore, these paths were removed from the modified model. The chi-square difference between the hypothetical model and the modified model was Δ*x*^2^(1) = 1.20, *p* > 0.05 and Δ*x*^2^(2) = 0.23, *p* > 0.05 for ethnic areas and non-ethnic areas, respectively. The hypothetical paths were verified using structural equation modeling and the BC bootstrap method (2,000 repetitions of sampling). The results of the operations are shown in Figures 2, 3 and Tables 5, 6. From Table 5, it can be seen that the fit of each model is basically acceptable. From Table 6, we can see that (1) in terms of the total effect, the psychological capital of teachers in both ethnic and non-ethnic areas has a significant positive predictive effect on career commitment. (2) In terms of direct effect, the direct effect of the psychological capital of teachers in ethnic areas on career commitment was significant, accounting for 37.04% of the total effect, while the direct effect of the psychological capital of teachers in non-ethnic areas on career commitment was not significant; the direct effect of the psychological capital of teachers in non-ethnic areas on career well-being was significant, accounting for 84.00% of the total effect of psychological capital on career well-being. (3) In terms of indirect effects, the mediating effect of career well-being on the psychological capital and career commitment of teachers in both ethnic and non-ethnic areas was significant, accounting for 62.96 and 85.14% of the total effect, respectively; the partially mediating effect of gratitude on psychological capital and career well-being for teachers in non-ethnic areas was significant, accounting for 14.67% of the effect of psychological capital on career well-being and the career well-being of teachers in non-ethnic areas. The fully mediated effect of gratitude on gratitude and career commitment was significant; the chain mediated effect of gratitude and career well-being on psychological capital and career commitment of teachers in non-ethnic areas was significant, accounting for 14.86% of the total effect. In summary, some of the hypotheses of this study were verified.

**TABLE 5:** Model fitness.

Grouping	Models	*x^2^/df*	Δ*x*^2^(Δdf)	CFI	NNFI	RMSEA	SRMR
Ethnic areas	Hypothesis model	3.64	1.20(1)	0.85	0.82	0.08	0.06
	Correction model	3.63		0.85	0.83	0.08	0.06
Non-ethnic areas	Hypothesis model	2.23	0.23(2)	0.89	0.87	0.09	0.07
	Correction model	2.19		0.9	0.87	0.08	0.07

**TABLE 6:** Total, direct, and indirect effects among the variables with the BC bootstrap test.

Paths	Ethnic areas			Non-ethnic areas		
	Standardized estimated value	*p*-value	95% CI	Standardized estimated value	*p*-value	95% CI
Psychological capital → Career commitment (total effect)	0.81	<0.001	[0.71 ∼ 0.88]	0.74	<0.001	[0.62 ∼ 0.85]
Psychological capital → Career commitment (direct effect)	0.3	<0.05	[0.08 ∼ 0.51]	–		
Psychological capital → Career commitment (total mediating effect)	0.51	<0.001	[0.37 ∼ 0.67]	0.74	<0.001	[0.62 ∼ 0.85]
Psychological capital → Career well-being (total effect)	–			0.75	<0.001	[0.64 ∼ 0.73]
Psychological capital → Career well-being (direct effect)	0.8	<0.001	[0.74 ∼ 0.87]	0.64	<0.001	[0.47 ∼ 0.79]
Psychological Capital → Gratitude → Career well-being	–			0.11	0.076	[0.02 ∼ 0.21]
Gratitude → Career well-being → Career commitment	–			0.23	0.083	[0.03 ∼ 0.47]
Psychological capital → Career well-being → Career commitment	0.51	<0.001	[0.37 ∼ 0.67]	0.63	<0.001	[0.46 ∼ 0.79]
Psychological capital → Career well-being → Gratitude → Career commitment	–			0.11	0.072	[0.02 ∼ 0.21]

## Discussion

### Analysis of the Characteristics of Teachers’ Psychological Capital, Gratitude, Career Well-Being and Career Commitment Levels in Ethnic Areas

This study investigated the basic conditions, relationships, and mechanisms of psychological capital, gratitude, career well-being, and career commitment for 402 primary and secondary school teachers in ethnic areas and another 171 teachers in non-ethnic areas. The findings indicated that the psychological capital of teachers in ethnic areas was slightly higher than that of teachers in non-ethnic areas, possibly because schools in ethnic areas are mostly located in more remote, non-central cities and have relatively poor conditions of school operation and social resources (Shao et al., 2018), given that this relative lack of external resources may cause teachers to rely more on internal psychological resources to cope with difficulties in their careers and lives. Taking into account teachers’ career commitment situations, there is no significant difference between the career commitment of teachers in ethnic areas and teachers in non-ethnic areas. That is, given similar levels of career commitment, teachers in ethnic areas required more psychological capital, suggesting that teachers in ethnic areas may require more psychological capital to maintain the same level of career commitment as other teachers. The results also revealed the importance of psychological capital for teachers in ethnic areas.

The examination of gender differences in psychological capital found that male teachers in ethnic areas had significantly lower psychological capital than female teachers, while there were no significant gender differences in the psychological capital of teachers in non-ethnic areas. First, the results concerning the difference in psychological capital of teachers in non-ethnic areas differ from those of certain previous studies, which concluded that male psychological capital is higher (Lehoczky, 2013; Liu, 2016). There is still much controversy concerning gender differences in psychological capital, and a meta-analysis of teachers’ psychological capital (Zhou and Zhou, 2017) had a small gender effect size (*g* = 0.067). This fact indicate that there is no substantial difference between the psychological capital of male and female teachers in general, while female teachers in ethnic areas have significantly higher psychological capital than male teachers. This result reflects the difference between the career characteristics and career psychological states of teachers in ethnic areas and those of other teachers. Teachers in ethnic areas had significantly lower career well-being than teachers in non-ethnic areas, which is a result of the objective conditions in ethnic areas (Cao, 2020). Some researchers have summarized the career challenges faced by teachers in ethnic areas as follows: lower remuneration; poorer working and living conditions; lower cooperation from parents and students; pressure on their own development; high workload; low social adaptation; and lack of social support, among others (Shao et al., 2018). Problems and limitations in these areas may make it more difficult for teachers in ethnic areas to experience satisfaction in their work and may cause their work to generate more negative emotions in their work, thus reducing the career well-being of teachers in ethnic areas.

The career well-being of female teachers in ethnic areas is significantly higher than that of male teachers, which is consistent with the findings concerning teachers in non-ethnic areas. This result indicates that female teachers in both ethnic and non-ethnic areas are more derive happiness from the teaching profession than their male counterparts. This difference may be because female teachers experience a greater sense of value in their educational work, and according to the perspective of gender roles proposed by Eagly et al. (2000), the internalization of masculine temperament (dominance, ambition, aggressiveness, etc.) make males more inclined to seek satisfaction and value in financial success and the acquisition of positions power. In contrast, the work of primary and secondary school teachers involved in this study is more repetitive and menial, which hardly fulfills the needs of the male gender role; instead, both the biological role of women and gender role tendencies (passion, compassion, etc.) make women more likely to obtain satisfaction and value from work related to children and adolescents, which leads to a generally higher career well-being for female teachers than for male teachers. The career commitment of female teachers in ethnic areas was significantly higher than that of male teachers. Based on the above gender role analysis, males more focused on career success than females, but the limitations of working conditions in ethnic areas make males more reluctant to sustain their teaching careers, while females focused on career stability more and thus relatively less willing to change careers.

Secondary school teachers in ethnic areas have lower levels of career well-being and career commitment than primary school teachers. Based on previous related survey data (Mou, 2018), this difference because that secondary school teachers in ethnic areas are faceing problems such as student dropout or pressure for further education, while lower career commitment caused by low career well-being. In terms of teaching subjects, teachers of political science, history, geography, science, and chemistry in ethnic areas have higher career well-being than teachers of language arts, mathematics, and physical education, while teachers of language arts and mathematics have higher career commitment than teachers of political science, history, geography, science, chemistry, and physical education; because that the necessity of teaching the “main subjects” has received more attention, so that these teachers have a relatively higher status. This result because that the necessity of teaching the “main subjects” is attributed more importance (Yuan, 2012) which has led to a relatively higher sense of career value and identity for these teachers and therefore a higher level of career commitment. This result also suggests that career commitment is influenced by objective conditions and environmental factors.

### The Relationship Between Teachers’ Psychological Capital and Career Commitment in Ethnic Areas, Its Significance, and Its Mechanism of Operation

The results of the study showed that psychological capital could significantly and positively predict career commitment, with a total effect of 0.81 for teachers in ethnic areas and 0.74 for teachers in non-ethnic areas, both of which had large effects. This result indicates that teachers’ psychological capital may indeed increase teachers’ career commitment to a great extent, which matches well with psychological capital theory. However, the direct effect of psychological capital on career commitment was significant only among teachers in ethnic areas and not among teachers in non-ethnic areas. This result is not fully consistent with the hypothesis of this study based on the internal motivation theory of Bandura and Locke (2003) suggesting that the mechanism underlying the effect of psychological capital on career commitment among teachers in non-ethnic areas may not operate through their own perceptions of competence to enhance internal motivation. In addition, the correlation analysis and structural equation modeling analysis showed a relatively large effect size for the psychological capital of teachers in ethnic areas. As mentioned above, this result because teachers in ethnic areas require more psychological capital, a subjective psychological resource, to maintain their career commitment in the face of additional practical difficulties, and this result, together with the above investigation of the basic profile of psychological capital and career commitment, points toward this conclusion. This result suggests that psychological capital may be of greater importance to teachers in ethnic areas than to teachers in non-ethnic areas. Unlike objective conditions such as employee social capital and human capital, which are usually referenced by previous management studies, psychological capital is an intrinsic psychological resource. Therefore, according to psychological capital theory and the work-resource model, when external resources or objective conditions are limited and difficult for the individual to change, the individual’s psychological capital becomes an important factor in supplementing the depletion of work resources and improving his or her organizational behavior. Future research can examine and explore this “compensatory role” of psychological capital further.

The mediation effect analysis showed that the mediation effect of career well-being on teachers’ psychological capital and career commitment in both ethnic and non-ethnic areas was significant, and this result was consistent with the hypothesis of this study, indicating that teachers’ psychological capital could indeed enhance their career commitment by enhancing their career well-being, which validated the extended and constructed model. Meanwhile, the mediating effect of teacher gratitude for teachers in ethnic areas was not significant, and this result was inconsistent with some of the hypotheses of this study. The mediation path diagram shows that although psychological capital can significantly and positively predict gratitude, gratitude cannot predict career commitment, and only among teachers in non-ethnic areas can gratitude act on career commitment through the full mediation of career well-being.

Among teachers in ethnic areas, gratitude could not significantly affect their career well-being. This result differs from the results of previous studies on the general well-being of individuals. This difference may be because the career well-being defined in this study primarily includes career identity, achievement satisfaction, and value realization, which is quite different from the ordinary sense of well-being (which focuses mainly on life satisfaction and positive emotions), which depends more on the individual’s self-fulfillment in his or her career and is a sense of eudemonic well-being (Ryan et al., 2008; Zhou et al., 2018; Wang et al., 2020). Meanwhile, it has been pointed out that the constraints of regional objective conditions in ethnic areas lower teachers’ career identity, value and achievement levels in ethnic areas (Shao et al., 2018), indicating that teachers’ career well-being in ethnic areas may also be constrained by many objective factors, which may also explain the fact that, while teachers’ gratitude in ethnic areas is positively associated with career well-being in the correlation analysis, in structural equation analysis, the predictive effect of gratitude was no longer significant when the effects of objective variables (e.g., gender, school level, etc.) were controlled for.

In summary, the hypotheses tested in this study were the predictive role of psychological capital on career commitment (Hypothesis 1) and the mediating role of career well-being on psychological capital and career commitment (Hypothesis 2). One hypotheses that could not be tested was the mediating role of gratitude on psychological capital and career commitment (Hypothesis 3). The sequential mediating role of gratitude on career well-being (Hypothesis 4) was tested only among teachers in non-ethnic areas but not among teachers in ethnic areas.

## Summary, Limitations, and Prospects

This study found that psychological capital had a significant positive predictive effect on teachers’ career commitment in both ethnic and non-ethnic areas, validating psychological capital theory. However, the direct effect of psychological capital on career commitment did not reach the significance level in non-ethnic areas. This result suggests that there are significant differences in the mechanisms by which teachers’ psychological capital works in ethnic and non-ethnic areas, and future research can explore the reasons for this fact further. Moreover, the effect of psychological capital in ethnic areas was slightly larger than in non-ethnic areas. This result is in line with the results of the mean comparison above. That is, teachers in ethnic areas require more psychological resources to maintain the same career commitment as other teachers. This fact suggests, on the one hand, that psychological capital is crucial to teachers in ethnic areas. On the other hand, it suggests that there may be some kind of compensatory mechanism that substitutes psychological capital for real resources. Future research can explore and verify this property of psychological capital from that perspective further.

In addition, psychological capital can act indirectly on career commitment through career well-being, validating the extended and constructed models. However, gratitude did not act directly on career commitment and could act indirectly on career commitment through enhanced career well-being only among non-ethnic teachers. This finding refutes the common speculation that perceived gratitude traits enhance employees’ gratitude toward the organization, increases employee loyalty, and thus enhances career commitment. A more plausible explanation is that gratitude traits enhance individual well-being, increase positive experiences at work, and thus enhance career commitment. Therefore, perhaps focusing on enhancing each teacher’s happiness and satisfaction is a more effective way of securing his or her career commitment as opposed to merely requiring teachers to be grateful and loyal to their profession.

There are certain shortcomings and limitations in this study that need to be supplemented and developed by future studies. First, this study tries to explain the effect of psychological capital on career commitment in ethnic areas from the perspective of gratitude and career well-being, but the effect of gratitude has not been verified in the sample of teachers in ethnic areas, which is inconsistent with the research hypothesis, and future research can analyze the reasons for this fact further through more empirical studies. Second, the sample of this study is mainly concentrated in Sichuan Province, which does not reflect the entire picture of ethnic areas in China. Future research can cover a wider range of ethnic areas to increase the diversity and representativeness of the sample. Finally, this study is only a cross-sectional survey study, and the causal relationship between variables needs to be verified further. Future research can verify further the causal relationship between variables through follow-up surveys and experimental studies (such as studies concerning the development of psychological capital).

## Data Availability Statement

The raw data supporting the conclusions of this article will be made available by the authors, without undue reservation, to any qualified researcher.

## Author Contributions

DH: the acquisition, analysis, or interpretation of data for the work and drafting the work or revising it critically for important intellectual content. TZ: substantial contributions to the conception or design of the work and final approval of the version to be published. KZ: the acquisition, analysis, or interpretation of data for the work, drafting the work or revising it critically for important intellectual content, and final approval of the version to be published. FD: drafting the work or revising it critically for important intellectual content. All authors contributed to the article and approved the submitted version.

## Conflict of Interest

The authors declare that the research was conducted in the absence of any commercial or financial relationships that could be construed as a potential conflict of interest.

## Publisher’s Note

All claims expressed in this article are solely those of the authors and do not necessarily represent those of their affiliated organizations, or those of the publisher, the editors and the reviewers. Any product that may be evaluated in this article, or claim that may be made by its manufacturer, is not guaranteed or endorsed by the publisher.

## Footnotes

1 Ethnic area is short for ethnic minority areas, which refer to areas where ethnic minorities mainly live together and implement the system of regional autonomy for ethnic minorities in these areas of China. At present, there are 155 national autonomous areas in China.
